# Assessing functional mitral regurgitation with exercise echocardiography: rationale and clinical applications

**DOI:** 10.1186/1476-7120-7-57

**Published:** 2009-12-14

**Authors:** Riccardo Bigi, Lauro Cortigiani, Francesco Bovenzi, Cesare Fiorentini

**Affiliations:** 1 Dipartimento di Scienze Cardiovascolari, Università degli Studi, Milano, Italy; 2 Divisione di Cardiologia, Ospedale "Campo di Marte", Lucca, Italy; 3 Dipartimento di Scienze Cardiovascolari, Università degli Studi e Centro Cardiologico Monzino IRCCS, Milano, Italy

## Abstract

Secondary or functional mitral regurgitation (FMR) represents an increasing feature of mitral valve disease characterized by abnormal function of anatomically normal leaflets in the context of the impaired function of remodelled left ventricles. The anatomic and pathophysiological basis of FMR are briefly analyzed; in addition, the role of exercise echocardiography for the assessment of FMR is discussed in view of its relevance to clinical practice.

## Functional mitral regurgitation (FMR)

**Figure 1:**
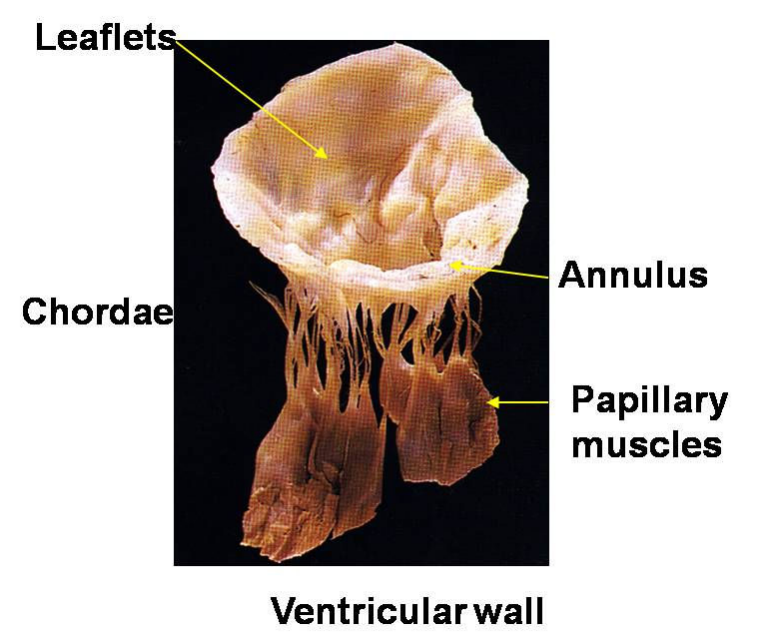
**The mitral valve apparatus**.

The mitral valve is a complex anatomical and functional unit including several components, namely mitral annulus, valve leaflets, chordae, papillary muscles and the underlying left ventricular (LV) wall (Figure 1). Normal function of the mitral valve apparatus depends on the anatomy of its components, three-dimensional relationship among them as well as LV size, shape and function.

Due to the regression of rheumatic disease and population aging with increasing degenerative or ischemic diseases, mitral valve disease has changed considerably during the past decades and is now represented mostly by pure or predominant mitral regurgitation.

Two different features of mitral regurgitation are traditionally described: a primary or organic and a secondary or functional (Table 1). Functional mitral regurgitation (FMR) is characterized by an abnormal function of normal leaflets in the context of an impaired LV function and is generally encountered in dilated and hypokinetic ventricles or as a result of a segmental damage of contractility. It occurs in 20-25% of patients after myocardial infarction [1-4] and up to 50% of those with heart failure [5]. However, it differs from the acute MR secondary to papillary muscle rupture.

**Table 1:** Classification of mitral regurgitation.

Primary (organic)	Mixomatous mitral valve disease
	
	Rheumatic disease
	
	Endocarditis
Secondary (functional)	Ischemic heart disease
	
	Dilated cardiomyopathy

The presence of FMR generally conveys an adverse prognosis as demonstrated by the lower survival rate associated with increasing values of Doppler-derived effective regurgitant orifice (ERO) [6]. In particular, a ERO >20 mm^2 ^has been reported to predict the worst outcome [6].

**Figure 2:**
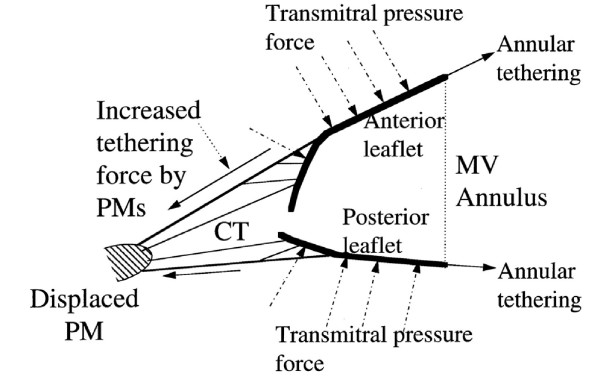
**Determinants of mitral valve dynamics**. See text for explanation.

**Figure 3:**
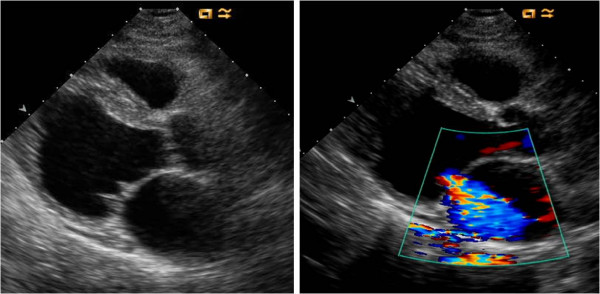
**Effect of LV remodeling on mitral valve dynamics**. See text for explanation.

**Figure 4:**
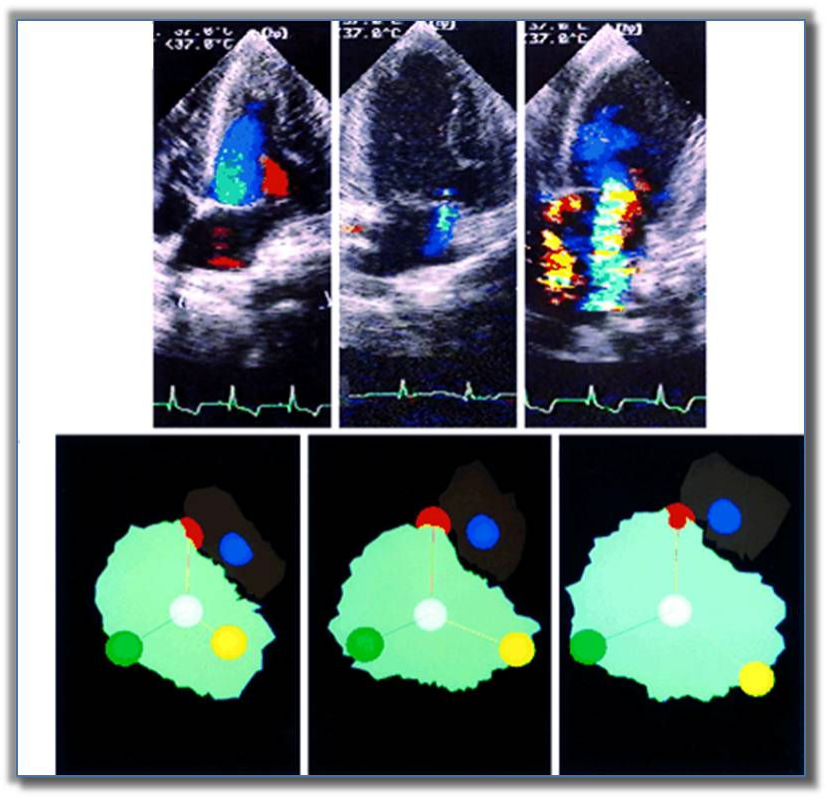
**End-systolic stop frame images and proximal flow-convergence region (A) at rest and during exercise in a patient with chronic inferior myocardial infarction and mitral regurgitation**. See text for explanation and comment). From ref [8]. Left: normal. Middle: LV dysfunction. Right: LV dysfunction and remodeling.

**Figure 5:**
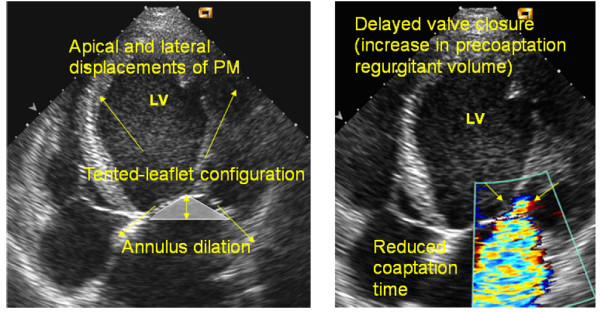
**Mechanisms of MR in dilated cardiomyopathy**.

During the systole, mitral valve dynamic is regulated by the balance between two opposite forces acting on the leaflets: the tethering force generated by the anulus and papillary muscles pulling the leaflets toward the apex and the closing force of the LV pushing the leaflets toward the atrium (Figure 2). Normally, just a little force is needed to close the leaflets. Therefore, an isolated contractile dysfunction causes just minor degree of MR [7]. Conversely, in case of LV remodeling, the myocardial segments underlying the papillary muscles bulge posteriorly and outward. The subsequent distorsion of the LV displace the papillary muscles which, in turn, pull the leaflets away from their normal coaptation and restrict their motion toward closure. This makes the tethering to overcome the closing force and leads to incomplete mitral leaflet closure (Figure 3). The additional effect of LV distorsion over the simple dysfunction has been demonstrated in experimental models [7] (Figure 4). In case of combined dilatation and remodelling (image on the right) the papillary muscles - shown as a yellow ball - migrate away and posteriorly from annular centroid (white ball) stretching the leaflets over larger annular area (pale green area) and producing more MR as compared to normal condition (left image) or LV dysfunction without remodelling (middle). The unifying view of FMR is summarized in the video clip [see additional file 1] simulating an ischemic remodelling of the LV, where the complementary role of dysfunction and distorsion in producing MR are clearly evident. Nevertheless, global hypoperfusion with LV dilatation, despite continued papillary muscles perfusion and thickening, can cause incomplete mitral leaflet closure and, finally, MR [8]. This is the case of dilated cardiomyopathy, universally associated with some degree of mitral regurgitation (usually mild to moderate) by creating spherical remodeling which affects the alignment of the mitral apparatus, dilates the annulus, and decreases the area of valve apposition (Figure 5).

## Effect of exercise on FMR

**Figure 6:**
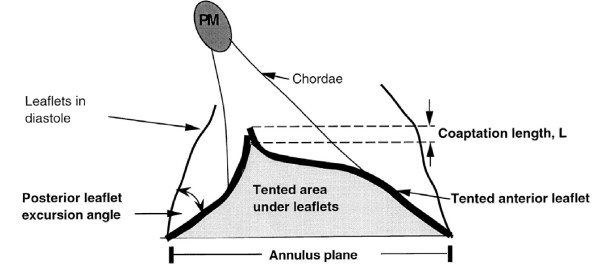
**Effect of the tenting area on ERO**. See text for explanation.

At rest, regurgitant volume depends on systolic pressure gradient across the orifice, duration of systole, and ERO dimension. During exercise, systolic pressure gradient increases, duration of the systole decreases, and regurgitant volume is strictly dependent on the size of ERO. Thus, FMR varies very differently from one patient to another during exercise and no correlation is observed with resting ERO [9]. Rather, the tenting area enclosed between leaflets and anular plane and the coaptation distance (the distance between annulus plane and coaptation point of the leaflets) become of critical importance in predicting exercise-induced changes in ERO (Figure 6). Lancellotti et Al. [9] found a strong correlation between changes in ERO and those in tenting area in 70 post-infarction patients with an ejection fraction <45%, at least mild MR at rest and no evidence of exercise-induced ischemia. More recently, Giga et Al. [10] performed exercise echo in 40 post-infarction patients with reduced ejection fraction and MR at rest. Of them, 78% showed an increase and 22% a decrease in MR during exercise. At univariable analysis, exercise-induced changes in MR were related to those in wall motion score index, sphericity index and different measures of mitral valve deformation (tenting area, coaptation distance and mitral anulus dimension). However, at multivariable analysis, just changes in coaptation distance and systolic tenting area were independent predictors of changes in ERO, thus confirming the pivotal role of mitral valve deformation.

It is important to note that exercise-induced changes in ERO can occur without evidence of myocardial ischemia. In the previously mentioned study [9], no patient had exercise-induced ischemia; nevertheless, 38 patients exhibited small and 19 larger increase in ERO, accounting for 81% of the studied population.

The mechanisms of exercise-induced changes in MR also relate to the site of previous myocardial infarction. The tenting area is the major determinant in patients with inferior, whereas the coaptation distance is the most powerful predictor in anterior infarctions [9]. In addition, a decrease in ERO during exercise occurs mainly in patients with inferior MI and recruitable viable myocardium [9]. Figure 3 provides a paradigmatic example showing the end-systolic stop frame images and proximal flow-convergence region at rest and during exercise in a patient with chronic inferior myocardial infarction and mitral regurgitation. During exercise, an evident contractile reserve of the basal inferior wall is recruited that is associated with a reduction in MR and PISA radius.

Main exercise-induced changes in FMR are summarized in Table 2.

**Table 2:** Characteristics of exercise-induced changes in FMR

1. greatly differ among patients
2. do not correlate with the degree of FMR at rest
3. do not correlate with LV dysfunction *per se*
4. mainly correlate with changes in mitral valve deformation
5. are more affected by local than global LV function and remodelling
6. are favourably affected by recruitable contractile reserve

## Technical considerations

Different echocardiographic approaches can be used for quantifying FMR. Semiquantitative methods, including the colour flow mapping of the regurgitant jet and the width of vena contracta are of limited value especially during exercise. Rather, quantitative Doppler echocardiography and the flow convergence or proximal isovelocity surface area (PISA) method seem to provide more accurate quantitation of FMR. A good correlation between the two methods has been demonstrated during exercise [11]. In case of appropriate flow convergence region, PISA represents the most reproducible and practical method, whereas the Doppler method can be an alternative in patients with a suboptimal flow-convergence definition. Changes in vena contracta width can be useful in patients with large exercise-induced increases of mitral regurgitant flow [11].

## Clinical relevance of exercise-induced changes in FMR

The effects of exercise on FMR have several implications which are relevant to clinical practice.

1) Exercise can unmask the severity of a seemingly mild MR.

**Figure 7:**
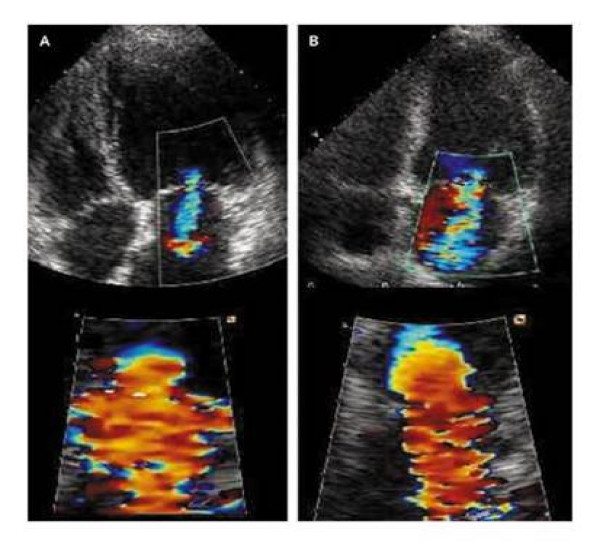
**Color-flow Doppler echocardiogram and the flow convergence proximal to the ERO at rest (panel A) and peak exercise (panel B) in a patient with nonischemic pulmonary edema**. From ref [10] A: rest. B: exercise.

Piérard et Al. [12] performed exercise echocardiography in 28 patients with LV dysfunction and recent acute pulmonary edema with no evidence of ischemia and 46 matched controls without a history of pulmonary edema. Despite similar features at rest, patients with pulmonary edema, unlike control subjects, doubled their regurgitant volume from mild to moderate-to-severe, with increased pulmonary pressures and limiting dyspnea (Figure 7). Thus, they appeared sensitive to the volume load imposed by the exercise and increase FMR.

2) Exercise-induced changes in FMR correlate with exercise capacity.

Lapu-Bula et Al. [13] studied 25 patients with mild-to moderate heart failure (NYHA class I-II) and LV dysfunction. According to the achieved peak VO_2_, 10 pts. had mild-to moderate (peak VO_2 _> 50% of age and sex-predicted value) and 15 severe (peak VO_2 _≤50% of age and sex-predicted value) exercise limitation. All measures of MR severity increased in almost every patient with exercise. However, the increase was statistically significant just in the group of patients with severe exercise intolerance.

3) Exercise-induced changes in FMR contribute explaining the origin of exercise limitation.

**Figure 8:**
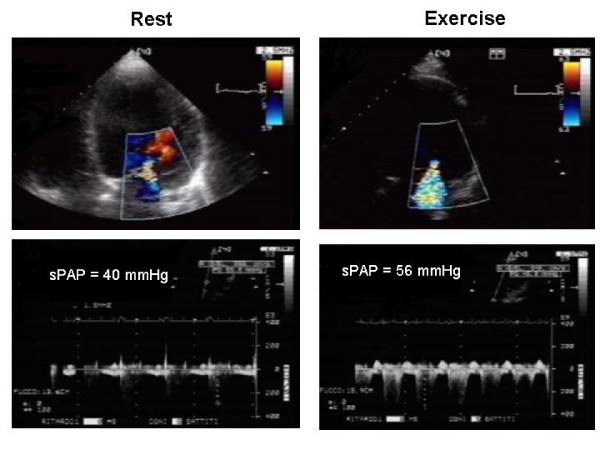
**Exercise-induced changes in mitral regurgitation and pulmonary arterial pressure in a patient with exertional dyspnea of unclear origin**.

Changes in MR observed during exercise have been demonstrated to inversely correlate with those in stroke volume [13] and directly with those in pulmonary wedge pressure [12] and transtricuspidal pressure gradient [11], thus providing a pathophysiological basis for interpreting the mechanisms underlying the reduced exercise tolerance observed in some patients (Figure 8).

4) Acute increase in FMR may cause nonischemic acute pulmonary edema.

Even though no correlation was found between ERO and transtricuspid pressure gradient at rest in patients with recent, nonischemic pulmonary edema, a significant correlation was demonstrated during exercise [12], thus suggesting that the dynamic nature of FMR may result in acute increase in pulmonary vascular pressure with major clinical correlates. This is further confirmed by the finding that exercise-induced changes in ERO along with transtricuspid pressure gradient and LV ejection fraction represent independent predictors of nonischemic pulmonary edema [10].

5) The relationship between contractile reserve during exercise and MR may have relevant therapeutical implications.

Differently from FMR related to segmental ischemia, MR secondary to increased sphericity and global dysfunction of the LV, as typically observed in dilated cardiomyopathy, is generally decreased by exercise-induced contractile reserve. This distinction has practical implications, as pure contractile dysfunction is expected to benefit from inotropic therapy, revascularization, or transplantation, whereas tethering might respond to modifying LV wall or papillary muscles geometry.

## Prognostic relevance

Exercise-induced changes in FMR also convey relevant prognostic implications.

No clinical data demonstrated a distinction between survivors and nonsurvivors among 98 consecutive patients with ischemic LV dysfunction and at least mild FMR who were prospectively followed up for 19 months [14]. However, Cox regression analysis indicated exercise-induced increase in ERO of at least 13 mm^2 ^as the most powerful predictor of cardiac death. In a more recent study [15], the same cut-off value was a significant predictor of cardiac death, hospital admission for worsened heart failure, and the combination of major adverse cardiac events. In addition, the increased transtricuspid pressure gradient and ERO >20 mm^2 ^emerged as predictors of mortality and hospital admission for heart failure and just cardiac mortality, respectively. Greater LV volumes at rest and lack of contractile reserve during exercise were additional independent markers of major adverse cardiac events.

## Conclusions

Substantial experimental and clinical evidence supports the use of exercise echocardiography for the assessment of FMR. In particular, it provides a useful tool to test the dynamic component of FMR and may unmask the real functional importance of what could be otherwise considered a mild-to-moderate dysfunction. In addition, it provides relevant prognostic information. Recent European Society of Cardiology guidelines on the management of valvular heart disease [16] include the use of echocardiography performed immediately after exercise as a potentially useful modality to assess the prognosis of MR, even though the need of conclusive findings, before this can be recommended in practice, is emphasized. On the other hand, the ACC/AHA 2006 guidelines [17] assign exercise echocardiography a class IIa indication (level of evidence C) in asymptomatic patients with severe MR to assess exercise tolerance and the effects of exercise on pulmonary artery pressure and MR. By a clinical standpoint, the question remains on which patient subgroups may benefit the most from performing the test. Based on the results of presently available studies, exercise echocardiography could be recommended as routine examination in some clinical settings:

1. patients complaining of dyspnea out of proportion to their LV dysfunction

2. patients with LV dysfunction and a history of pulmonary edema with no evident origin

3. for the prognostic assessment of patients with FMR

4. patients with FMR candidates to surgical revascularization for evaluating the opportunity of a combined approach with mitral surgery

5. for the selection of the optimal surgical approach

**Figure 9:**
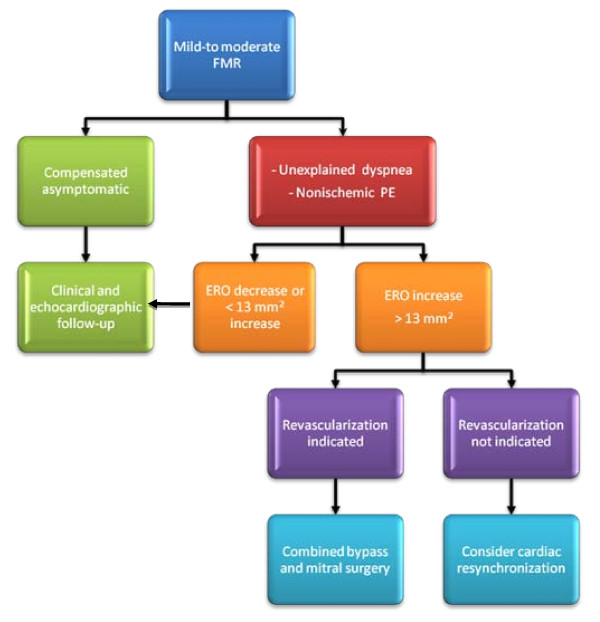
**Algorithm of clinical use of exercise echocardiography**. FMR: functional mitral regurgitation. PE: pulmonary edema. ERO: effective regurgitant orifice.

A possible diagnostic algorithm of clinical use of exercise echocardiography is reported in Figure 9.

## Future developments

New parameters available from exercise echocardiography may contribute refining the usefulness of the technique for assessing FMR. The force-frequency relationship (FFR), a noninvasive index of global contractility easily determined during exercise echocardiography, has been proposed as an adjunctive tool for identification of limited contractile reserve and latent global left ventricular dysfunction [18,19]. The presence of regional differences in the heart rate dependence of contractility has been demonstrated in LV tissue obtained from human hearts with chronic MR [20]. This regional FFR variation is expected to correlate with diastolic shape changes associated with remodeling during chronic MR, thus grounding the use of FFR analysis in conjunction with exercise echocardiography as an adjunctive tool for the diagnostic and prognostic assessment of FMR.

## Competing interests

The authors declare that they have no competing interests.

## Authors' contributions

RB conceived of the study, performed bibliographic research and drafted the manuscript. LC participated in the study design and coordination. FB and CF contributed critical revision of the manuscript. All authors read and approved the final manuscript.

## Supplementary Material

Additional file 1 **Effect of ischemic remodelling of the LV**. Video animation showing the complementary role of dysfunction and distorsion in producing FMR. Click here for file
